# The SS18-SSX fusion oncoprotein: Friend and foe in targeted therapy for synovial sarcoma

**DOI:** 10.32604/or.2025.060573

**Published:** 2025-04-18

**Authors:** GAVIN M. ANCHONDO, KYRA PARKER, ALEXIS BRUCE, ELIZABETH CORTEZ, LE SU

**Affiliations:** Department of Biology, Jacksonville State University, Jacksonville, AL 36265, USA

**Keywords:** SS18-SSX, Synovial sarcoma, Targeted therapy

## Abstract

Synovial sarcoma is a high-grade soft tissue malignancy characterized by a unique fusion gene known as SS18-SSX. The SS18-SSX fusion protein acts as an oncogenic driver of synovial sarcoma, and it has thus been commonly accepted that disruption of SS18-SSX function represents a therapeutic means of treating synovial sarcoma, but emerging evidence suggests that upon depletion of SS18-SSX, an anti-apoptotic signal surprisingly arises to protect synovial sarcoma cell survival. In this article, we discuss the controversial roles of SS18-SSX’s transcriptional activity in synovial sarcoma biology and outline a synergistic strategy for overcoming the resistance of synovial sarcoma cells to SS18-SSX targeted therapeutics.

## Synovial Sarcoma

Synovial sarcoma is a rare type of cancer that generally affects about 1000 individuals in the United States a year but has a high incidence among children and young adults [1]. The origin of synovial sarcoma is believed to develop from mesenchymal progenitor cells in the soft tissue surrounding the feet, neck, extremities, and appendages [2]. Its histological features include monomorphic spindle cells with variable epithelial differentiation, whilst rarely are poorly differentiated tumors made up of small round cells with enlarged nuclei [3]. The SS18-SSX fusion gene is a specific and reliable diagnostic marker for synovial sarcoma due to its unique occurrence in this cancer type [4]. Aside from SS18-SSX, high levels of the transcriptional corepressor named Transducin-like Enhancer of Split 1 (TLE1) is specifically found in most, if not all, of synovial sarcoma tumors and is thereby used as an additional way to indicate the presence of synovial sarcoma in the clinic [5]. However much of the problem with identifying synovial sarcoma in an individual results from little or unnoticed symptoms at an early stage. In this regard, the primary cancer may have already spread before it is diagnosed, especially considering that synovial sarcoma has a high rate of metastasis with 50% to 70% of cases [6–9]. Treatment for synovial sarcoma is complex: not all treatment options work for every individual. Surgical amputation or resection of the affected anatomy is the most conventional way to combat this type of cancer. Radiation and chemotherapy may also be prescribed for synovial sarcoma patients. Radiotherapy is heavily dependent on the tumor size; the denser the tumor the more radiation is needed at the location of the affected tissue. Synovial sarcoma has also been reported to be susceptible to some chemotherapeutic reagents such as anthracycline and ifosfamide [10,11]; however, there is an aversion to this method, as synovial sarcoma largely afflicts children and teenagers. Most often the survival rate of synovial sarcoma patients is based on a 5-year period, varying from 50% to 60% [9,12]. Conversely, the returning metastasis rate increases after the third year of initial diagnosis despite treatment methods in place. For patients with metastatic synovial sarcoma, it has been estimated that the median survival is only 16–24 months [9,13].

## The Mechanism of SS18-SSX Action

The fusion gene SS18-SSX is a mutation that occurs after the reciprocal translocation t(X;18)(p11;q11) between chromosomes 18 and X. The two separate genes SS18 and SSX1/2 (rarely, SSX4) fuse together and encode a chimeric protein mostly comprised of the original SS18 sequence while also gaining a new carboxy-terminus with 78 amino acids of the SSX region [14]. Wild-type SS18 protein is a major subunit of the SWItch/Sucrose Non-Fermentable (SWI/SNF) complex, which typically activates gene expression via maintaining an open and accessible status of chromatin [15]. By contrast, wild-type SSX protein interacts with Polycomb repressive complexes (PRCs), resulting in chromatin condensation and inhibition of transcription [16]. It is of interest that the SS18-SSX fusion protein preserves the transcriptional regulatory functions of both SS18 and SSX. On one hand, SS18-SSX acts as a transcriptional corepressor, replacing wild-type SS18’s role in gene activation with certain SSX-associated repressive molecules such as TLE1, histone deacetylases (HDACs) and PRCs [17–21]. On the other hand, this fusion protein also enables SWI/SNF complex assembly at specific PRC-bound genomic loci, thereby increasing the accessibility of nearby chromatin and causing aberrant activation of target genes [22,23]. Notably, many of the genes dysregulated by SS18-SSX have important functions in controlling cell growth and differentiation [14,19]. This is consistent with the prior finding that expression of the SS18-SSX fusion gene in a noncancerous context (such as mesenchymal progenitor and myoblast cells) can impair normal skeletal muscle development and promote aberrant cell proliferation, contributing to synovial sarcoma-like tumor formation in transgenic mice [21,24,25]. In light of these findings, SS18-SSX has been generally considered an appealing therapeutic target for synovial sarcoma treatment.

## Targeted Therapeutics for Synovial Sarcoma

Targeted therapy that aims to block the oncogenic pathways driven by SS18-SSX in particular through disruption of its complex with transcription regulators is an active area of synovial sarcoma research. For example, Bromodomain Containing 9 (BRD9) is crucial for the assembly of SS18-SSX-associated SWI/SNF complex in synovial sarcoma cells, and suppression of BRD9 by chemical degraders has been shown to attenuate tumor growth in a synovial sarcoma xenograft mouse model [26]. However, an obvious limitation of this approach is that BRD9 is required only for SS18-SSX-mediated transcriptional activation but not for gene repression. Indeed, transcriptomic studies indicated that targeting BRD9 could perturb only a very limited subset of SS18-SSX target genes in synovial sarcoma cells [26]. Another druggable target is Enhancer of Zeste 2 (EZH2), which functions as a methyltransferase to establish repressive histone methylation signals at certain SS18-SSX target loci, resulting in gene silencing [17,18]. Consistent with this view, blocking EZH2 activity with small molecules (such as EPZ005687 and tazemetostat) was reported to induce the removal of repressive histone marks and subsequent activation of some tumor suppressor genes usually repressed by SS18-SSX in synovial sarcoma cells [27,28]. While cell line-based xenograft studies yielded further evidence for the anti-synovial sarcoma activity of EZH2 inhibitor, inconsistencies in drug efficacy were seen among the mouse models implanted with primary tumor tissues from different synovial sarcoma patients [28]. Consistent with this finding are the clinical studies that showed that high levels of EZH2 are only associated with a proportion of synovial sarcoma patients [29,30], suggesting caution on EZH2 inhibitor implication in the clinical setting. Collectively, these observations reflect the challenges of “killing two birds with one stone” in targeting SS18-SSX-driven mechanisms, which involve a dual role in both upregulation and downregulation of target gene expression, different from other sarcoma-associated fusion oncoproteins acting monotonously as either activators or repressors.

While direct inhibitors of SS18-SSX are lacking, recent studies have identified histone deacetylase (HDAC) inhibitors (for instance, FK228, SB939, and quisinostat) as promising agents that disrupt SS18-SSX function in two different ways. One mechanism of HDAC inhibitor action occurs at the transcriptional level and works by dissociation of the TLE1 corepressor from chromatin to restore normal gene expression disrupted by SS18-SSX [18,31,32]. The second mechanism relies on a series of post-translational events, marking SS18-SSX for protein degradation [33,34]. Specifically, HDAC inhibitor treatment acetylates Murine Double Minute 2 (MDM2), a ubiquitin ligase that normally catalyzes the destruction of another ubiquitin ligase named Mcl-1 Ubiquitin Ligase E3 (MULE). Acetylation of MDM2 then blocks its interaction with MULE, thus releasing MULE to ubiquitinate SS18-SSX for proteasome-dependent protein breakdown. These observations support using HDAC inhibitors as an attractive anti-synovial sarcoma strategy, which provides an advantage of attacking the oncogenic driver SS18-SSX, rather than an individual cofactor or a selected downstream target. In light of these mechanistic findings, some HDAC inhibitors have entered clinical studies to treat patients with synovial sarcoma and other advanced sarcomas. While modest clinical benefit was seen in some patients, the overall efficacy of HDAC inhibitors as monotherapy has proved not to be enough to control disease progression [35]. In a phase II trial, for example, the best evaluable response after a maximum of six cycles of SB939 treatment was stable disease (2 of 3 synovial sarcoma patients and 7 of 15 other sarcoma cases) [36]. This clinical outcome highlights the need for an improved understanding of the SS18-SSX function in synovial sarcoma biology and raises an interesting possibility that SS18-SSX induces synovial sarcoma development but is not indispensable for synovial sarcoma cell survival.

## Negative Feedback between SS18-SSX Depletion and Therapeutic Resistance

An important clue comes from a CRISPR/Cas9-modified synovial sarcoma cell model, in which a FLAG epitope is tagged to the carboxy-terminal end of endogenous SS18-SSX protein [33]. Using the anti-FLAG antibody, a set of proto-oncogenes that are targeted directly by SS18-SSX have been identified by chromatin immunoprecipitation (ChIP)-based sequencing analysis [37]. Many of these genes appear to be expressed in an SS18-SSX-dependent manner, in line with the traditional thinking that SS18-SSX establishes an oncogenic program driving synovial sarcoma development. But one exception is the proto-oncogene, FYN, which shows increased expression in synovial sarcoma cells after SS18-SSX depletion. This is a contentious finding because FYN encodes a Src family protein kinase known to induce anti-apoptotic signaling in many cancers by dysregulation of Ak strain transforming (AKT) activity [38]. In this case, disrupting SS18-SSX function may potentiate FYN kinase activation which, in turn, protects synovial sarcoma cell survival. In fact, the elevated levels of FYN have been found in response to HDAC inhibitor-induced SS18-SSX protein degradation, and blocking FYN kinase activity with the small molecule PP2 can substantially enhance (about 10- to 30-fold) the sensitivity of synovial sarcoma cells to the clinically-applicable HDAC inhibitor FK228 in a dose-dependent fashion [37]. Importantly, this synergistic effect is not limited to synovial sarcoma cell lines, but is also true for primary patient cells, in 2D and 3D culture models. Nevertheless, it is worth noting that truly “selective” inhibitors for FYN do not yet exist. Indeed, PP2 was shown to target both FYN and another Src family kinase, lymphocyte-specific kinase (Lck) [39], the latter of which has the known function of promoting cancer cell proliferation and chemoresistance [40,41]. Since the role of Lck in synovial sarcoma biology remains understudied, further investigation will be required to clarify the relevance of Lck kinase activity to SS18-SSX-targeted therapy. A detailed analysis of the Lck signaling pathway may yield additional candidate molecules that can be targeted by pharmacological agents to overcome HDAC inhibitor-induced resistance in synovial sarcoma and potentially other related sarcomas.

## Conclusion

**Figure 1:**
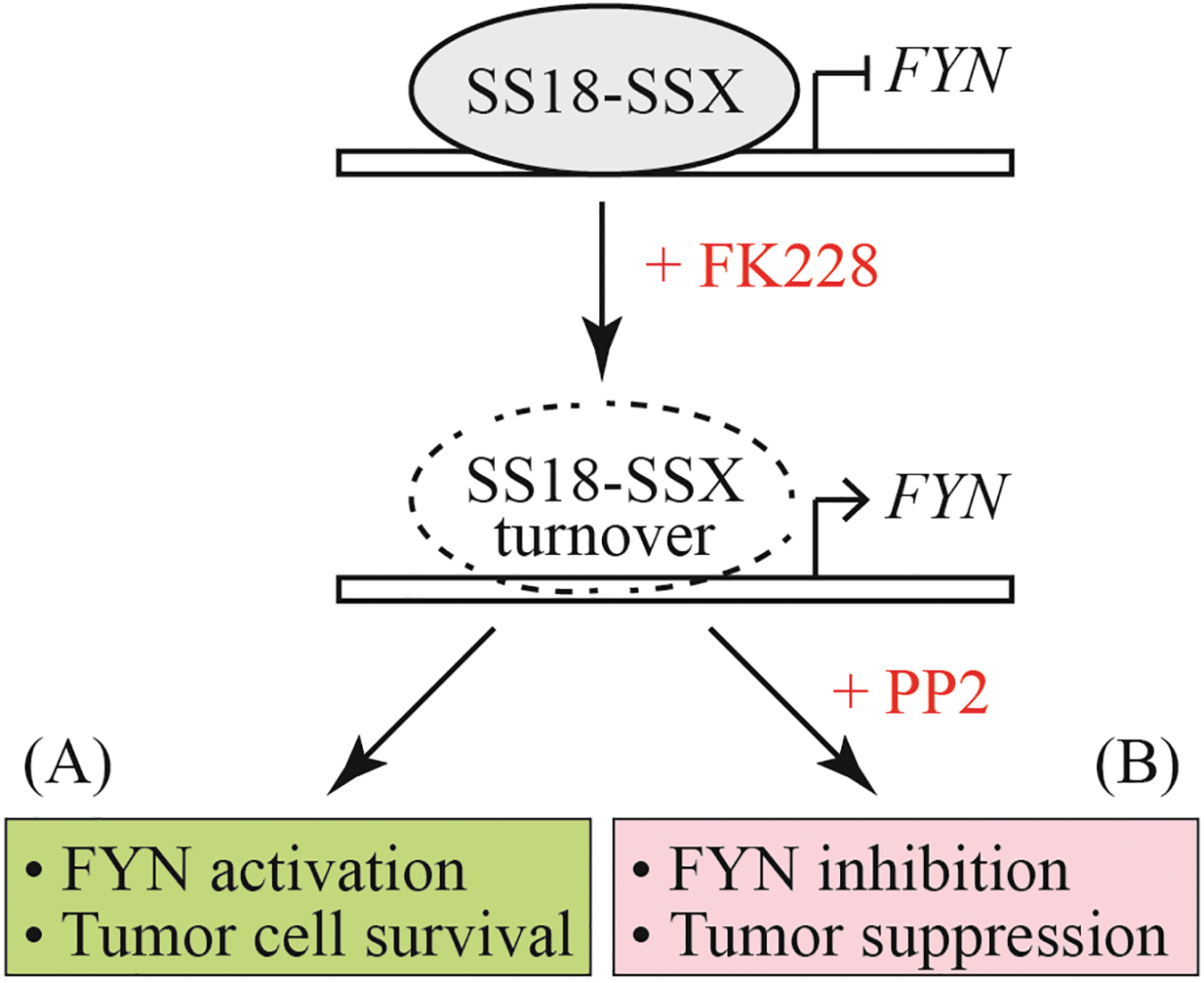
A model for SS18-SSX control of FYN gene expression and comparison of HDAC inhibitor monotherapy *vs*. combination therapy in synovial sarcoma. (A) SS18-SSX suppression by HDAC inhibitor (FK228) treatment activates the expression of its target gene FYN promotes sarcoma cell survival and potentially causes drug resistance. (B) Co-treatment of synovial sarcoma cells with HDAC and FYN inhibitors (FK228 + PP2) can not only disrupt the SS18-SSX function but also diminish FYN activity to overcome therapeutic resistance [37]. The dashed cycle represents SS18-SSX protein degradation.

In summary, we outline a new mode of SS18-SSX action in synovial sarcoma therapy (schematically shown in Fig. 1). The SS18-SSX fusion protein has been widely accepted as a proactive oncoprotein, which mostly operates as part of a transcription regulatory complex permitting aberrant activation of proto-oncogenes and therefore contributing to cellular transformation. We now know that SS18-SSX also has a “self-protective” function that is not involved in synovial sarcomagenesis, but instead sustains synovial sarcoma cell survival upon loss of SS18-SSX. The best evidence for this view of SS18-SSX function is that genetic or pharmacologic inhibition of SS18-SSX reactivates its target gene FYN and promotes FYN protein accumulation in synovial sarcoma cells. FYN is an essential regulator of the AKT signaling pathway, which commonly participates in abnormal cell proliferation and therapeutic resistance in many cancers [42]. If so, FYN upregulation may enable synovial sarcoma cells to survive without the need for the presence of SS18-SSX. This would, at least in part, explain the observed inefficiency of HDAC inhibitor treatment in clinical trials for synovial sarcoma. Notably, FYN itself is a druggable target [39], and exposure of synovial sarcoma cells to the small molecule PP2 (a potent FYN inhibitor) brings about the enhanced efficacy of HDAC inhibitor treatment. Given these observations, it seems that despite a “cause-and-effect” relationship between SS18-SSX and synovial sarcoma, suppression of SS18-SSX alone could accidentally evoke certain oncogenic events (such as FYN activation) that must be monitored closely for achieving effective therapy. Indeed, a recent report suggested that higher levels of FYN protein are associated with chemo-resistance in ovarian cancer patients and that depletion of FYN significantly resensitizes ovarian cancer cells to platinum-based chemotherapy [43]. Given the ability of SS18-SSX to negatively regulate FYN expression, it would not be surprising, if FYN activation in response to SS18-SSX-targeted therapy served as a poor prognostic factor for synovial sarcoma. From a broad perspective, similar logic might also be extended to other synovial sarcoma-like sarcomas by virtue of a defining translocation event; in particular, the fact that many translocations generate fusion transcription factors acting as a hub to regulate a large and diverse set of target genes in a network makes one wonder whether their depletion by targeted therapy would also stimulate as-yet-undefined “self-protective” mechanisms that elicit the undesirable effects of negative feedback and therapeutic resistance.

## Data Availability

Not applicable.

## Abbreviations

AKT: Ak strain transforming
BRD9: Bromodomain containing 9
EZH2: Enhancer of zeste 2
HDAC: Histone deacetylase
Lck: Lymphocyte-specific kinase
MDM2: Murine double minute 2
MULE: Mcl-1 ubiquitin ligase E3
PRC: Polycomb repressive complex
SWI/SNF: Switch/Sucrose Non-Fermentable
TLE1: Transducin-like enhancer of split 1
